# Laboratory Methods for Assessing the Influence of Improper Asphalt Mix Compaction on Its Performance

**DOI:** 10.3390/ma13112476

**Published:** 2020-05-29

**Authors:** Michał Wróbel, Agnieszka Woszuk, Wojciech Franus

**Affiliations:** Department of Geotechnical Engineering, Faculty of Civil Engineering and Architecture, Lublin University of Technology, Nadbystrzycka 40, 20-618 Lublin, Poland; m.wrobel@pollub.pl (M.W.); a.woszuk@pollub.pl (A.W.)

**Keywords:** asphalt mix, compaction index, volumetric parameters, stiffness modulus, moisture resistance

## Abstract

Compaction index is one of the most important technological parameters during asphalt pavement construction which may be negatively affected by wrong asphalt paving machine set, weather conditions, or the mix temperature. Presented laboratory study analyzes the asphalt mix properties in case of inappropriate compaction. The reference mix was designed for AC 11 S wearing layer (asphalt concrete for wearing layer with maximum grading of 11 mm). Asphalt mix samples used in the tests were prepared using Marshall device with the compaction energy of 2 × 20, 2 × 35, 2 × 50, and 2 × 75 blows as well as in a roller compactor where the slabs were compacted to various heights: 69.3 mm (+10% of nominal height), 66.2 mm (+5%), 63 mm (nominal), and 59.9 mm (−5%) which resulted in different compaction indexes. Afterwards the samples were cored from the slabs. Both Marshall samples and cores were tested for air void content, stiffness modulus in three temperatures, indirect tensile strength, and resistance to water and frost indicated by ITSR value. It was found that either insufficient or excessive level of compaction can cause negative effect on the road surface performance.

## 1. Introduction

The quality of an asphalt road surface depends on many factors, including: The type of materials used (aggregates, asphalt, and others), climatic conditions, traffic load, the drainage system, and the method of construction [1,2]. Important factors directly determining the service life of the road surface are the features of the designed asphalt mix, such as: fatigue limit, resistance to rutting, or resistance to water and frost [3,4,5]. Other parameters, such as the density of the mineral-asphalt mixture or the air void content (porosity), are indirect. It is generally assumed that in case of dense-graded asphalt mix the content of air voids should be high enough to avoid rutting and low enough at the same time to limit water and dirt penetration into the surface structure [6]. In addition, the surface texture is related to the content of air voids in the wearing layer, which has a direct impact on the safety of road users [7]. Hence, the need for appropriate compaction of particular pavement layers has been known since the beginning of asphalt road construction technology. The main purpose of asphalt mix compaction is to achieve appropriate density to meet the requirements for physical and mechanical properties and to ensure a tight surface which will be exploited for the maximum possible life cycle [8]. It is a difficult process involving the selection of appropriate compaction parameters in order to achieve the assumed compaction index (CI) of the layer.

Under laboratory conditions, samples of asphalt mix can be compacted in various ways, depending on the purpose of the sample (type of tests), the applicable regulations, as well as the type of technology used [9]. The most commonly used devices are gyratory compactor, Marshall compactor, asphalt roller compactor, and a vibrating compactor [10]. These methods differ in many parameters, such as the pressure force, the way the force is transmitted, compaction time, and also the final shape of the obtained sample and the particles orientation inside [11]. In many European countries the basic method is Marshall compaction method which involves dropping the compacting hammer on a sample of asphalt mix in cylindrical form a certain number of times, which depends on the further use of the sample. Another way is to use a gyratory compactor, where the sample is compacted to the required density or a certain number of rotations, while being simultaneously compacted and sheared [12]. The method is essential in the case of projecting asphalt concrete mixes for very thin layers (BBTM). Moreover, A. Woszuk stated that in order to determine the operating temperatures of Warm Mix asphalt the gyratory compaction method is recommended [10]. According to the European standards [13], the sample placed in a cylindrical form with a diameter of 100 mm or 150 mm is subjected to a constant compressive stress of 600 kPa during the rotational movement, which is transmitted through the piston to parallel sample bases. During the compaction, the sample is rotated at a speed of 30 rpm and the angle of inclination is 1.25° (typical parameters in the Superpave system [14]). Such process better reflects the compaction in real conditions than Marshall method as well it is possible to determine significant coefficients concerning susceptibility to compaction of the mix: compaction coefficient K, mixture stability index MSI, and mixture resistance index MRI [15,16]. Georgiou et al. found that in the case of compacting in the gyratory device, the samples were segregated, whereas in the case of cores they had a low level of segregation [17]. Additionally, in order to obtain the internal structure of samples comparable to roller compaction, the compaction angle parameter should be appropriately selected, slightly higher than that specified by technical regulations. Taking into account the resistance to rutting, Tapkin and Keskin demonstrated that the samples made in the gyratory press were less susceptible to permanent deformations than those compacted using the Marshall method [18]. Lee et al. found that in case of crumb rubber asphalt mixes air void content was significantly dependent on the number of gyratory rotations and directly related to this rutting increased along with air voids [19]. In reference to the interlayer bonding, Jaskuła showed that the higher the compaction index, the higher was maximum shear strength between the layers [20]. The shearing and mechanical behavior is one of the major issues in many other fields like food industry, paints and coating industry, and also energy industry. In these areas hybrid gels featuring interpenetrating covalent can be applied, which exhibit both high toughness and recoverability of strain-induced network damage [21].

In the rutting test, however, samples made in a roller compactor are used [22]. Airey and Collop indicate that slab-compacted specimens are more closely correlated with cores sampled from the road surface than gyratory and vibratory-compacted specimens [11]. There are also self-compacting mixes, e.g., mastic asphalt, often used in bridge structures [23] or SCC cement concrete [24].

Under real construction conditions, the target compaction is carried out by road rollers of different sizes and types of drums, however the first compaction is carried out by the paver. A vibrating and heated board (table) levels and pre-compacts the mix and shapes longitudinal and transverse slopes. This is a very important process as it allows to achieve about 90% of the required compaction of the pavement layer. In order to effectively develop the road surface design, the compaction of the mixture in the laboratory must properly simulate the compaction in real conditions [25].

According to the European standard, the required level of density expressed as percentage refers to the volumetric density obtained for samples made in the Marshall compactor compared to the results obtained for samples cored from the pavement. The degree of compaction of the layer on site, in addition to the testing of the cores, can also be determined by nuclear and non-nuclear density gauges [26]. In relation to these types of density tests, Micaelo et al. noted that the nuclear method has greater fluctuations in results [27]. Another method may be the density measurement based on an analyzer using neural networks which has the ability to predict the density in real time during the compaction process [28,29].

During road construction (also in laboratory) there is a risk of improper layer compaction, which results from several factors: inappropriate choice of rollers, weather conditions, properties, and temperature of the asphalt mix. Praticò et al. also stated that volumetric properties are also influenced by the test method and even the diameter of the samples [30].

As shown in the presented literature analysis, relevant compaction significantly affects the quality of the asphalt road surface. Moreover, literature data clearly indicate that there are strong correlations between the compaction method and the mechanical properties of asphalt mix. The main aim of this article is to assess the influence of improper compaction (including under or over-compaction) on the quality and durability parameters of the road surface such as density, air void content, stiffness modulus, indirect tensile strength, and resistance to water and frost.

## 2. Experimental Materials

### Asphalt Mix Components

The bitumen used in the tests was 50/70 asphalt from ORLEN Asfalt Sp. z o.o. (Plock, Poland). The properties of asphalt binder are presented in Table 1.

**Figure 1:**
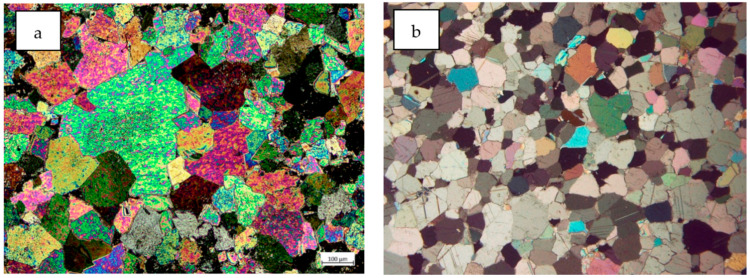
Microphotographs of dolomite (**a**) and limestone (**b**).

**Figure 2:**
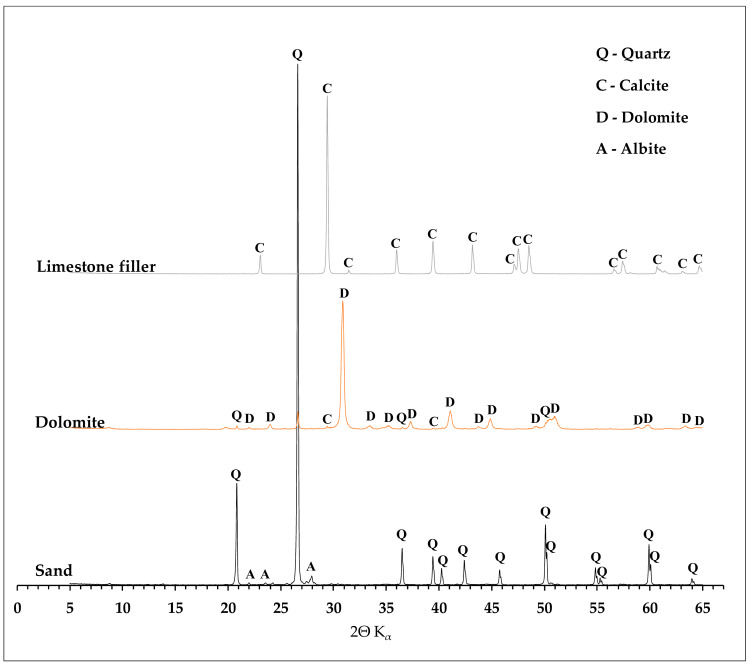
Mineral composition of used aggregates and filler.

**Figure 3:**
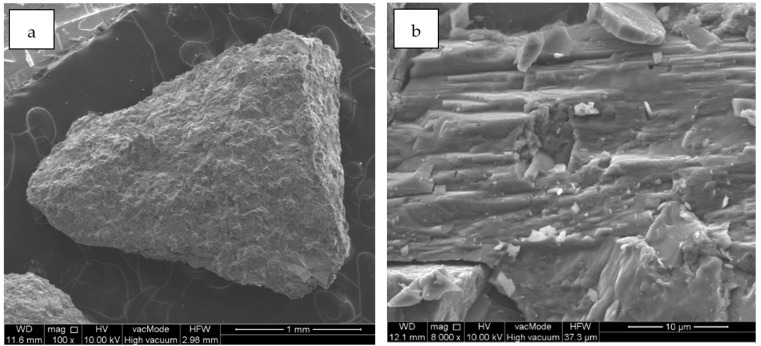

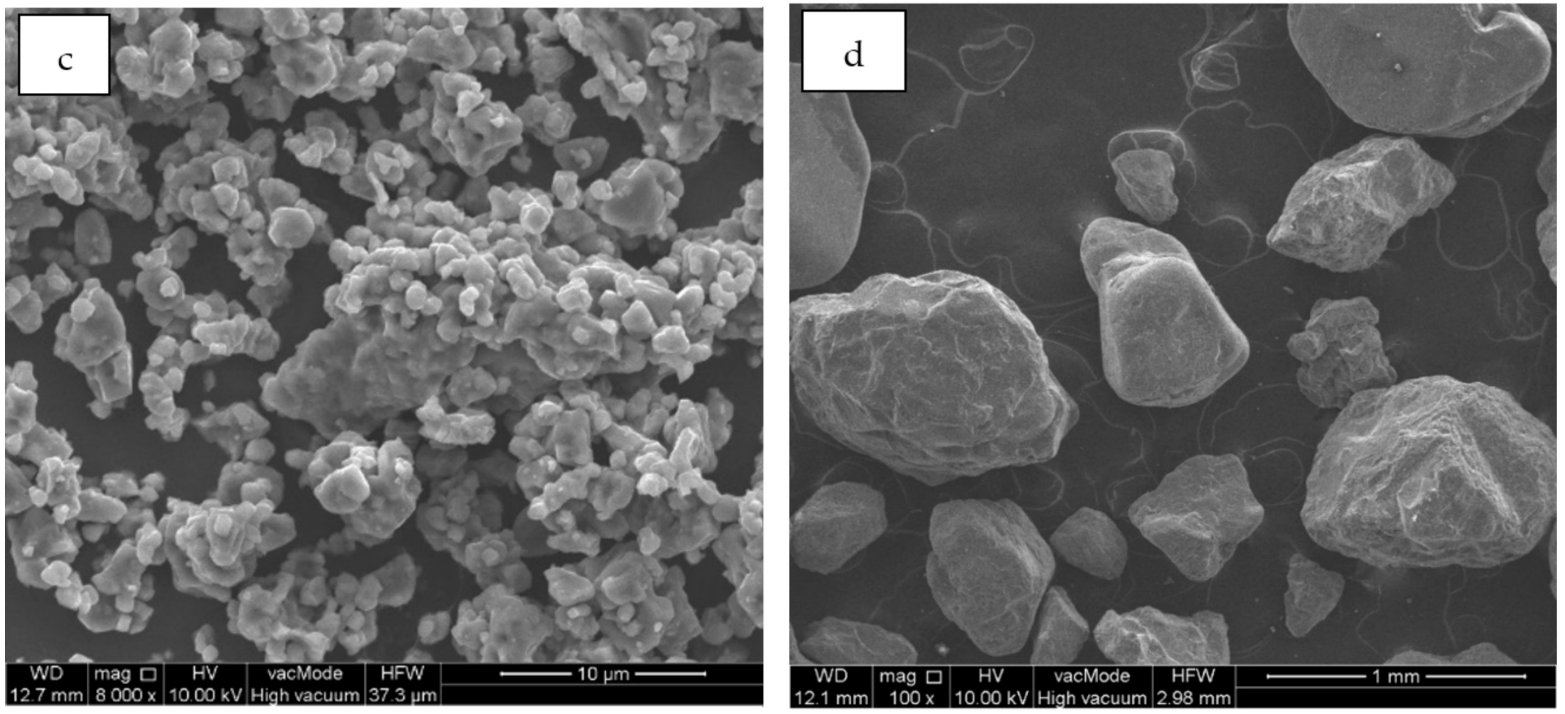
Scanning electron microscopy (SEM) images of dolomite (**a**,**b**), limestone filler (**c**) and sand (**d**).

The aggregates used in laboratory tests were sand, dolomite, and limestone as a mineral filler. The dolomite used in the research comes from the Piskrzyn deposit (Kopalnie Dolomitu S. A.) while limestone from the Bukowa deposit (Lhoist Bukowa Sp. z o.o.). Both mines are located in the Świętokrzyskie Province (Poland). The sand used in the study comes from the Baranówka deposit. The microstructure of the initial carbonate raw materials (dolomites and limestone) used in the study is presented in Figure 1. The chemical composition of the minerals determined by the X-ray fluorescence spectrometry (XRF) method is presented in Table 2. For carbonate raw materials, the dominant chemical component is CaO, which is 80.94% for limestone filler and 39.62% for dolomite. In the case of the latter, MgO is also an important component, with a content of 14.76%. For quartz sand the main component is SiO_2_ (92.99%). Silica is also present in dolomite aggregate and its content is 10.05%. The remaining chemical components in the tested raw materials are present in minor quantities. Mineral composition of used aggregates and filler identified using the X-ray diffraction (XRD) method is presented in Figure 2. The content of individual mineral phases was determined by the Rietveld refinement. For dolomite aggregate, the content of dolomite equals 87.6%, 1.2% of calcite and 11.2% of quartz. In case of limestone filler the calcite content is 98.3%. The mineral composition of the filler is supplemented with quartz in the amount of 1.7%. In the sand used for the study, the main component is quartz, which is 93.6%, followed by marginal amounts of potassium feldspar identified as albite (6.4%). SEM images of the materials used are shown in Figure 3. The shape of dolomite grains is shown in Figure 3a and can be described as angular, while the character of their surface is presented in Figure 3b and can be clearly marked by a three-way cleavage which results in rough character. Limestone filler and crushed sand are presented in Figure 3d respectively. The limestone grains reach a size of about 1μm and represent a fairly regular shape. Quartz grains present in the sand most often reach irregular shapes emphasized by a clear fracture.

Aggregate grading was determined by dry sieving method [31]. In case of the filler air jet sieving test was applied [32]. The composition of the mineral mix was designed using the grading envelope method applicable in Poland [22].

**Figure 4:**
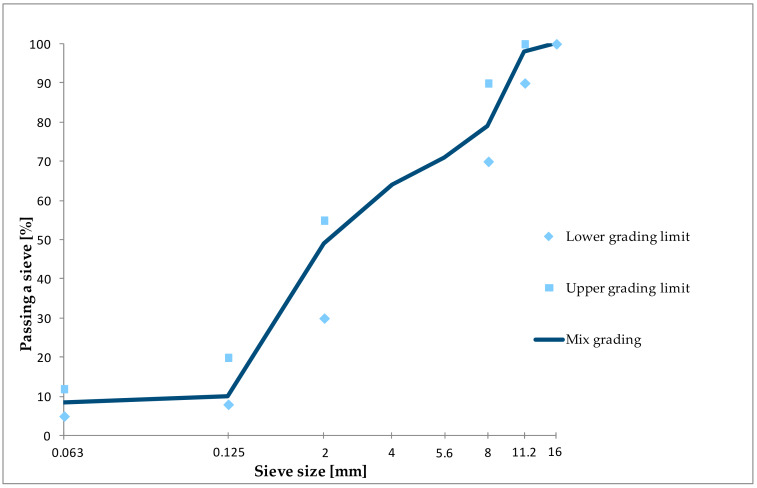
Grading of the mineral mix.

The designed reference asphalt mix is intended for construction of asphalt concrete wearing layer (AC 11 S) on roads with traffic loads of KR category 1–2 in accordance with Polish technical standards [22]. The content of dosed asphalt was 5.7% in relation to the weight of the asphalt mix. Figure 4 shows the grading of the mineral mix. The composition of both mineral and asphalt is presented in Table 3.

## 3. Research Method

### 3.1. Samples Preparation

Samples made of reference asphalt mix were compacted in a Marshall device using 50 blows per side and named M3. The compaction temperature was 135 °C according to Polish requirements [19]. Samples were also compacted using different energies: 20, 35, and 75 blows per side, named M1, M2, M4 respectively. In order to reflect the compaction method in the real conditions prevailing during the placement of asphalt mix into the road surface and to assess the influence of the method on the properties samples were also made in a slab roller compactor that uses tilting pivoted circular sector with closed loop control system. To achieve various compaction indexes, samples were compacted to variable target heights: 63 mm, 66.2 mm, 69.3 mm, and 59.9 mm. The weight of the batch used for each slab was the same and was calculated taking into account the density of the asphalt mix, assuming that a 63 mm high slab sample should have a compaction index of 98% which is the minimum value required for compacting the asphalt mix under real conditions.

The attempt to produce a sample with a height of 56.7 mm (reduced by 10% compared to 63 mm) failed. The compaction device stopped at a slab height of 58.0 mm and the obtained sample was deformed, thus excluded from further testing.

Cylindrical cores were cut out of each slab for further testing and marked with symbols:

- Cores sampled from slab no. 1, 69.3 mm high (63 mm + 10%)—R1
- Cores sampled from slab no. 2, 66.2 mm high (63 mm + 5%)—R2
- Cores sampled from slab no. 3, 63 mm high—R3
- Cores sampled from slab no. 4, 59.9 mm high (63 mm − 5%)—R4

### 3.2. Test Methods

Volumetric parameters:

Compaction index was determined on core samples according to the following formula:

$$CI=\frac{\rho_{core}}{\rho_{Marshall}}\times 100\;\left(\%\right)$$

where:

CI—compaction index [%]
ρ_core_—bulk density tested on core sample
ρ_Marshall_—bulk density determined on Marshall samples

Bulk density was determined using the B method—saturated surface dry (SSD) [33] and calculated for each sample according to the formula:

$$\rho_{bssd}=\frac{m_{1}}{m_{3}-m_{2}}\times \rho_{w}$$

where:

*ρ*_bssd_—bulk density (SSD) (kg/m^3^)
*m*_1_—mass of the dry specimen (g)
*m*_2_—mass of the specimen in water (g)
*m*_3_—mass of the saturated surface-dried specimen (g)
*ρ*_w_—density of water at test temperature (kg/m^3^)

Maximum density was determined experimentally according to the EN 12697:5:2018 standard [34] and calculated using the following formula:

$$\rho_{m}=\frac{m_{2}-m_{1}}{1000\times \left(V_{pp}-\frac{m_{3}-m_{2}}{\rho_{w}}\right)}$$

where:

*ρ*_m_—density of the asphalt mix (kg/m^3^)
*m*_1_—mass of the pycnometer (g)
*m*_2_—mass of the pycnometer with the specimen (g)
*m*_3_—mass of the pycnometer with the specimen and water (g)
*ρ*_w_—density of water at test temperature (kg/m^3^)
*V*_pp_—Volume of the pycnometer filled to the measuring line (m^3^)

Air void content was calculated according to the EN 12697-8:2018 [35] using the following formula:

$$V_{m}=\frac{\rho_{m}-\rho_{b}}{\rho_{m}}\times 100\%$$

where:

*V*_m_—air void content in the asphalt mix specimen (%)
*ρ*_m_—density of the asphalt mix (kg/m^3^)
*ρ*_b_—bulk density of the asphalt mix (kg/m^3^)

ITSR:

Water and frost resistance tests of the asphalt concrete were performed based on the EN12697-12:2018 standard [36]. This test evaluates the effect of one freeze-thaw cycle of saturated mix asphalt samples on indirect resistance to stretching. Both the samples made in the Marshall compactor and the samples cut from the slabs were divided into two sets. The control series samples were conditioned in a laboratory on flat surface in room temperature of 20 ± 5 °C. The samples from the second set were conditioned in water at 40 °C for 72 h, then frozen at −18 °C for 16 h and re-conditioned in water at 25 °C for 24 h. After conditioning, the indirect tensile strength of all test pieces was tested according to EN 12697-23 at 25 °C. Based on the obtained results, an indicator of resistance ITSR was calculated:

$$ITSR=\frac{ITS_{w}}{ITS_{d}}$$

where:

ITSR—indirect tensile strength ratio (%)
ITSw—the average strength for the wet set of samples (kPa)
ITSd—the average strength for the dry set of samples (kPa)

Stiffness modulus:

The tests of the stiffness modulus were performed according to the EN 12697-26:2018 standard [37]. Controlled force was applied to each sample. The samples were subject to five dynamic loads applied to the sample vertically, along the diameter of the base. Force increase time, measured from zero to a maximum value, was 0.124 s. Maximum force generated a horizontal dislocation of sample equal to 5 µm. The sample, after performing the test, was rotated by 90° around the horizontal axis and tested again. A reliable stiffness modulus for each sample was a mean average out of two measurements. The final result was the arithmetic mean out of three tested samples. The tests were performed in three temperatures: 23 °C, 10 °C, −2 °C. The following Poisson’s ratios were applied for the temperatures, respectively: 23 °C–0.4; 10 °C–0.3; −2 °C–0.25.

## 4. Results and Discussion

### 4.1. Bulk Density and Compaction Index

**Figure 5:**
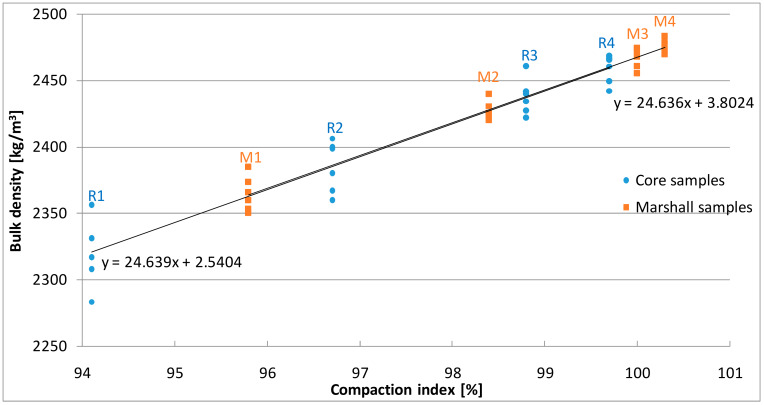
The results of bulk density.

The results of bulk density of the tested samples are presented in Figure 5.

Bulk density of the reference samples was 2467 kg/m^3^. The results for Marshall samples varied from 2364 kg/m^3^ to 2475 kg/m^3^. The core samples cut from the slabs are characterized by bulk density ranging from 2321 kg/m^3^ to 2459 kg/m^3^. In Figure 5, a linear decrease in bulk density can be observed as the slab height increases. A similar trend can be observed for Marshall samples under decreasing compaction energy. The average results of bulk density were the basis for determining the compaction indexes listed in Table 4.

According to the Polish technical regulations, the compaction index of asphalt concrete during the paving process is at least 98% [38]. On the other hand, slabs prepared in the laboratory for testing resistance to permanent deformations should have a compaction index ranging from 98% to 100% [22]. According to the assumptions, a change in the target height of slab compaction influenced the compaction index value. Samples cut from slabs with a height reduced by 5% (59.9 cm) had a compaction index of 99.7%. This value is within the requirements for both laboratory samples for further testing and for mixtures compacted with rollers during road surface preparation. Slabs of increased thickness were characterized by compaction index at the level of 97.3% and 94.3%, which indicates under-compaction of the mixture. Insufficiently compacted asphalt layers are more susceptible to deeper water penetration and more intensive asphalt oxidation and, as a consequence, to faster surface degradation. On the contrary, excessively compacted asphalt pavements are more susceptible to permanent deformations and low-temperature cracking [39].

The Marshall samples compacted using 50 blows per side are reference samples with a compaction rate of 100% in accordance with Polish requirements. As expected, the compaction index was dependent on the compaction energy. However, as in the case of the slab compactor, this parameter was found to be not very susceptible to compaction. Although similar correlations of density and compaction index were obtained. Proper compaction of the tested mixture is significantly influenced by the applied filler with regular grain shapes. As indicated by Zulkati et al. if the filler has a large enough diameter and a regular shape, it acts as a friction-lubricate agent. This facilitates a faster and smoother reorientation movement of larger aggregates, thereby resulting in high compaction susceptibility [40]. Conversely, Melloti et al. noticed that irregular shaped filler has a negative effect on the workability of asphalt mix [41].

As shown by previous studies, particle orientation and general aggregate structure is significantly different in samples compacted by different methods. Slab samples are characterized by an even particle size distribution across, while samples produced using other methods of laboratory compaction are susceptible to circumferential particle orientation. Contact and interlocking of aggregates, depending on the shape of the aggregate, also has a significant impact on the compaction. Use of angular aggregate particles means achieving more contact points and more uniform distribution of internal forces, with a better interconnection between elements and improvement to fatigue performance as well as permanent deformation resistance [42]. In the tests, crushed aggregates were used, which in combination with the asphalt binder guarantee the durability of the created bonds. Moreover, applied aggregates have an angular shape, which was confirmed by SEM analysis. Finally, the orientation and segregation of aggregates affects both the air void content and the mechanical properties of the asphalt mix [11].

### 4.2. Density And Air Void Content of the Asphalt Mix

**Figure 6:**
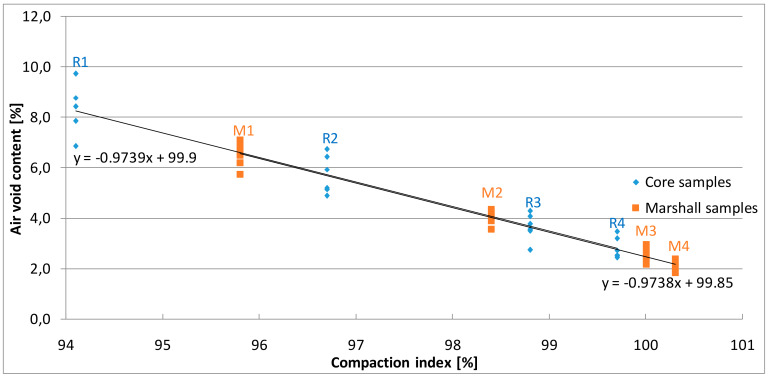
The results of air void content calculations.

The results of the air void content calculations are presented in Figure 6.

Air void content was calculated according to the maximum density of the asphalt mix that was 2530 kg/m^3^ and the bulk density of individual samples. The values of the parameter determined on Marshall samples varied from 2.2% to 6.5% and was adequate to the number of blows (Table 4). The air void content of AC 11 S reference samples made was 2.5%. According to technical regulations [19], asphalt concrete can be used for the wearing course if the samples’ porosity is between 1 and 3%. The analysis of the obtained results shows that increasing the compaction energy above the standard requirements results in a slight change in the content of air voids; with an increase in compaction by 50% (from 50 × 2 to 75 × 2), a decrease in voids was from 2.5% to 2.2% and the compaction index was 100.3%. On the other hand, the reduction in the number of blows during the compaction of samples results in a considerable increase in free spaces. As indicated by Lucas Júnior et al. in samples with high air void content, the mutual blocking of aggregates is not fully possible because of their shape and texture. As a consequence, the functional properties of the produced mix deteriorate [43].

The content of air voids determined for samples cut out of slabs was from 2.8% to 8.3%. The value increased with the decrease of the compaction index, which is the expected relation. The core samples with a 99.7% compaction index were characterized by a free space content of 2.8%, which was closest to the results obtained for reference samples. It was also noted that in samples with the 98.8% compaction index, the free air void content was above the upper limit according to Polish requirements and was 3.7%, however the requirement of 1 to 3% porosity refers to samples made using the Marshall method. In case of surfaces made of AC 11 S mix compacted with rollers in real conditions, the technical recommendations [38] indicate that this layer should be compacted at a rate above 98% and contain between 1 and 4.5% air voids. It can be concluded that the samples made in a roller compactor with a compaction rate of at least 98% meet the requirement for the porosity of the asphalt layer. Nevertheless, the results of these tests are based on the assumed simulation of compaction by rolling in the laboratory, and the differences in the results are the effect of different slabs thicknesses. Under real conditions, the correct compaction of each layer is expected. In addition, one of the important elements influencing the proper effect of this process is the temperature of the asphalt mix—the lower the larger the voids in the layer, which is confirmed by both volumetric studies [16,44] and x-ray computed tomography analyses [45]. Wang L et al. noticed that not only the percentage content of air voids affects the performance of the surface, but also the spatial distribution and pore diameter [46]. Jiang W et al. proved that the characteristics of air voids are significantly influenced by aggregate gradation [47]. Moreover, the structure of aggregate, number of contact points, and orientation of each aggregate are dependent on the compaction methods and conditions [48].

### 4.3. Water and Frost Resistance

**Figure 7:**
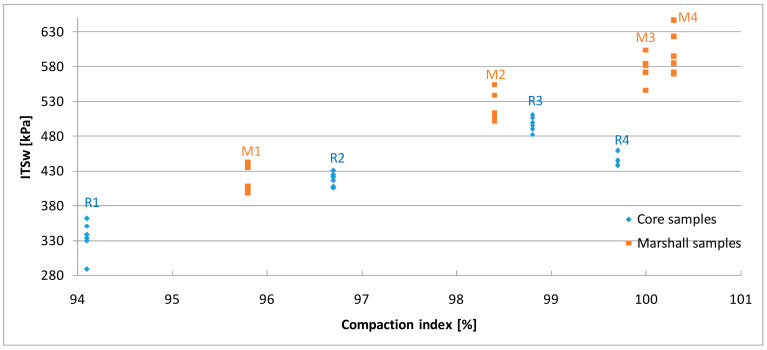
The results of indirect tensile strength test for the wet set.

**Figure 8:**
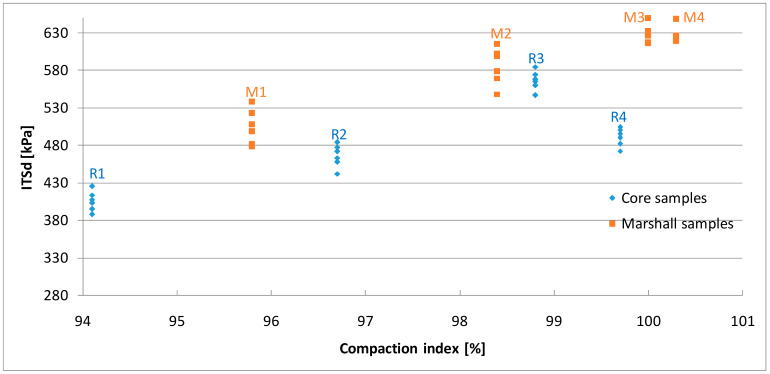
The results of indirect tensile strength test for the dry set.

The results of the indirect tensile strength test are shown in Figure 7; Figure 8 as well as Table 5. The strength obtained for the samples compacted using Marshall method was in the range of 504 kPa to 648 kPa in the dry set and 421 kPa to 598 kPa in the wet set. It was observed that the results were dependent on the compaction energy, whereas in the case of 2 × 75 blows the strength increased only by 3% in the dry set and 4% after freezing cycle comparing to the reference samples.

Considering the core samples in general, it was noted that the strength increased with the growth of the compaction index, however the strength of the samples with the highest compaction index (99.7%) turned out to be lower than expected. This is probably due to the over-compaction of the asphalt mix. A further consequence of excessively “dense” mixtures is a decrease in resistance to permanent deformations and low-temperature cracking [39]. It was also observed that asphalt mix has reduced strength characteristics if the air void content is above the required limits. This is particularly the case for R1 samples where the porosity was the highest (8.3%) and the strength characteristics were lowest, 406 kPa for dry set and 334 kPa after the freezing cycle respectively. The acceptable limit of voids content was also exceeded in R4 samples, which is reflected in their strength.

Correlations between the compaction methods and the obtained strength results were observed. At a similar value of compaction index Marshall samples were more resistant than cores, in a similar range from 54 kPa to 91 kPa on average in dry set and from 33 kPa to 87 kPa after freezing cycle. The difference may be caused by change in aggregate interlock pattern in the particular method [49]. Thus, the strength of the samples is directly related to the compaction process, which significantly affects all properties of the asphalt mix.

The results of water and frost resistance tests are presented in Table 4. The average ITSR parameter for reference Marshall specimens was 91.7%. In case of the other Marshall samples the value varied from 83.5% for the least compacted to 92.3% for the most compacted ones. R4 core samples, which on the basis of the test results were classified as over-compacted had high resistance to water and frost of 91.2%. The same was observed for most dense Marshall samples. This is probably the effect of very low air void content limiting water absorption in a natural way, which resulted in a slight decrease in strength of samples subjected to one freeze-thaw cycle. As predicted, the lowest ITSR value was obtained on R1 samples that were much under-compacted and had the highest air void content. Thus, moisture damage appeared, which is associated with both loss of cohesion and loss of adhesion [50]. In reference to the nominal value of Marshall compaction energy, R3 samples was less frost resistant than expected, even though they were properly compacted and had the highest strength among the samples cut out of slabs. However, in tests of asphalt mix properties there is not always a positive correlation between strength and frost resistance measured by ITSR [51,52].

As is well-known, moisture damage resistance of asphalt mix is strongly related to the aggregate-binder adhesion. Among the aggregates used, dolomites with CaO content of 39.62% prevailed. Alkaline aggregates are characterized by excellent adhesion to asphalt, which results not only in high water resistance but also in good strength parameters. Lucas Júnior et al. indicates that the indirect tensile strength also depends on aggregate sphericity and texture [43].

The mixture also contains filler with a very high lime content. It is naturally hydrophilic material with a tendency to form strong bonds with hydrophobic organic compounds such as bitumen [53].

### 4.4. Stiffness Modulus

**Figure 9:**
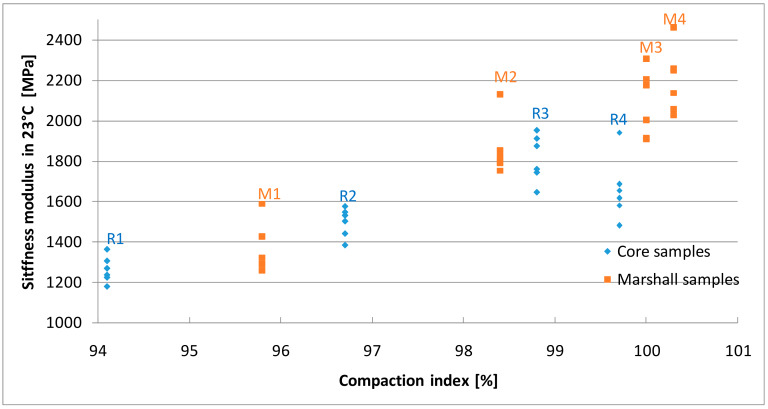
The results of stiffness modulus tests in 23 °C.

**Figure 10:**
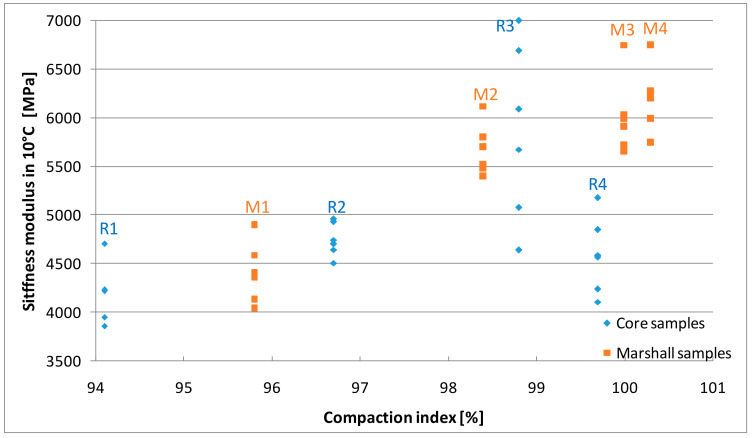
The results of stiffness modulus tests in 10 °C.

**Figure 11:**
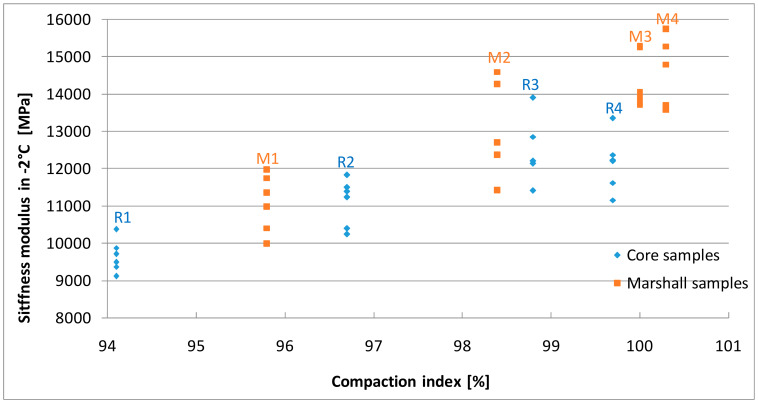
The results of stiffness modulus tests in −2 °C.

The results of stiffness modulus tests are presented in Figure 9, Figure 10 and Figure 11 and Table 6. The values of the parameter determined on the Marshall samples varied from 1384 MPa to 2197 MPa in 23 °C, 4404 MPa to 6201 MPa in 10 °C, 11,075 MPa to 14,446 MPa in −2 °C. Compaction index was found to have a linear correlation to the increasing stiffness modulus.

The stiffness of the samples cut out of the slabs increased as the density index increased, but only to a certain level. In case of 99.7% compaction index core samples, results were 1660 MPa in 23 °C, 4590 MPa in 10 °C, and 12162 MPa in −2 °C and were lower than the values obtained for samples with 98.8% CI value. These results confirm previous assumptions that R4 samples with the highest compaction index (99.7%) were over-compacted, whereas in case the Marshall samples compacted with increased energy a decrease in stiffness did not occur. Moreover, for similar compaction indexes, samples made using the Marshall method had higher stiffness than samples cored from slabs, which is consistent with the research of Hartman et al. [54]. This may be due to different compaction energies and the size and type of mold in the individual methods [11].

High stiffness modulus asphalt mixes are also characterized by higher resistance to rutting at positive temperatures [55]. Based on the analysis of the test results obtained, it can be concluded that over-compacted mixtures not only have lower strength but are also less resistant to high temperatures, including the development of permanent deformations. At negative temperatures, on the other hand, higher values of the asphalt mix stiffness modulus are expected.

Asphalt changes its properties as a result of oxidation processes and, as time passes, asphalt surfaces become even stiffer and consequently more susceptible to low temperature cracks and premature degradation [39,56]. Therefore, controlling the stiffness of the designed asphalt mix is a factor that can prevent early defects of the surface. It is particularly important in the case of uncertain compaction process and adaptation of additives softening the asphalt binder [57,58,59].

In the conducted research the core samples that occurred to have the lowest stiffness in −2 °C (9661 MPa) are characterized by the lowest compaction index (94.3%), the highest porosity (8.3%), and the lowest frost resistance (ITSR = 82.4%).

The results clearly indicate that the stiffness modules tend to decrease as the voids increase, which is the expected relation [9,56].

## 5. Conclusions

The research presented in the article concerned the assessment of the influence of improper compaction of mineral-asphalt mixtures determined by the compaction index on the properties of the produced mix, and in particular on the resistance to water and frost. The studies were carried out on samples compacted using Marshall method with different compaction energies as well as on samples cut from slabs which were compacted to different target heights. The mineral-asphalt mix was an asphalt concrete designed for the wearing course of an asphalt pavement—AC 11 S.

The analysis of the obtained results indicates that inappropriate compaction of asphalt mixes is reflected in the results of physico-mechanical properties. The core samples with the required compaction index of at least 98% also had an air void content in the range corresponding to the technical recommendations for asphalt pavement layers, i.e., from 1 to 4.5%. However, the free space content increased as the compaction index decreased, which is in line with expectations.

As a general rule, compared to core samples, Marshall samples were characterized by higher indirect tensile strength and stiffness modulus at similar compaction index values. Although the Marshall method is very popular, it does not reflect compaction on the construction site well due to the vertical-only impact movement, in contrast to the roller compactor. Despite the 50% increase in compaction energy, the Marshall samples also do not exhibit features typical of excessively compacted samples such as reduced strength and stiffness.

The results of the obtained tests indicate that under-compacted asphalt mixes are characterized by the lowest indirect tensile strength both in dry state and after soaking as well as the lowest stiffness modulus regardless of the test temperature. Also the ITSR value was the lowest for these samples, which confirms that insufficiently compacted asphalt layers are more vulnerable to water and frost. The increased air voids content allows water molecules to penetrate deeper, which combined with the freeze-thaw cycles, causes premature degradation of the pavement.

When compacting asphalt mixes, over-compaction is particularly dangerous. As indicated by the analysis of the results, the compacted mix may have a compaction index and air void content within the required limits. However, there may be a loosening of the aggregate and damage to the contact points, which results in reduced resistance to weather conditions leading to the destruction of the road surface. Also the analysis of the test results indicates a decrease in strength and stiffness for over-compacted core samples.

In the compaction analysis, the properties of the aggregates used should also be taken into account, such as grain shape, surface character, or mineralogical composition. Crushed aggregates with a rough surface and regular grain shapes ensure proper mutual interlocking and a durable connection on the contact with the asphalt binder.

In order to identify the primary cause of a change in the physico-mechanical properties of abnormally compacted mineral-asphalt mixtures, it is necessary to analyze the damage at the contact between aggregate and asphalt as well as conduct computer simulations including finite element analysis.

## Figures and Tables

**Table 1:** Properties of the base bitumen.

Test	Specification	Result	Specification Limit
Penetration (25 °C, 0.1 mm)	EN 1426:2009	59	50–70
Viscosity at 135 °C (mPa·s)	ASTM D 4402	428	-
Softening point(°C)	EN 1427:2009	53	46–54
Penetration index	EN 12591:2010	−0.1	-

**Table 2:** Chemical composition of the studied materials.

	Dolomite (% wt)	Limestone Filler (% wt)	Sand (% wt)
MgO	14.76	0.21	-
Al_2_O_3_	3.53	0.14	2.80
SiO_2_	10.05	0.25	92.99
K_2_O	1.42	-	0.82
CaO	39.62	80.94	0.25
TiO_2_	0.24	-	0.09
MnO	0.23	-	-
Fe_2_O_3_	2.15	0.07	1.29
LOI	28.00	18.39	1.78

**Table 3:** Composition of mineral mix and asphalt mix.

Material Type	Percentage (%)	
	Mineral Mix	Asphalt Mix
Mineral filler	6.0	5.5
0/2 Quartz, fine aggregate	20.0	18.8
0/2 Dolomite, fine aggregate	26.0	24.6
2/8 Dolomite, coarse aggregate	26.0	24.6
8/11 Dolomite, coarse aggregate	22.0	20.8
50/70 Asphalt	-	5.7

**Table 4:** Specimen determination, bulk density results, air void content results, and compaction index values.

Specimen Type	Height Change (%) Compaction Energy (Blows)	Bulk Density (kg/m^3^)	Standard Deviation	Air void Content (%)	Standard Deviation	Compaction Index (%)
R1	+10 *	2321	25	8.3	1.0	94.1
R2	+5 *	2385	19	5.7	0.8	96.7
R3	0 *	2437	14	3.7	0.5	98.8
R4	−5 *	2459	11	2.8	0.4	99.7
M1	2 × 20 **	2364	5	6.5	0.5	95.8
M2	2 × 35 **	2427	8	4.1	0.3	98.4
M3	2 × 50 **	2467	7	2.5	0.3	100.0
M4	2 × 75 **	2475	13	2.2	0.2	100.3

* Height change of the slab samples (%); ** Compaction energy of Marshall samples (Blows)

**Table 5:** Average results of indirect tensile strength for the wet set and standard deviations.

	Compaction Index (%)	ITS_w_ (kPa)	Standard Deviation	ITS_d_ (kPa)	Standard Deviation	ITSR (%)
Cored samples	94.1	334	25	406	13	82.4
	96.7	418	10	466	15	86.6
	98.8	497	10	567	13	87.8
	99.7	448	10	491	12	91.2
Marshall samples	95.8	421	19	504	23	83.5
	98.4	520	22	585	24	88.9
	100	577	19	629	12	91.7
	100.3	598	30	648	33	92.3

**Table 6:** Average results of stiffness modulus in and standard deviations.

	Compaction Index (%)	Stiffness in 23 °C (MPa)		Standard Deviation	Stiffness in 10 °C (MPa)	Standard Deviation	Stiffness in −2 °C (MPa)	Standard Deviation
**Cored Samples**	94.1	1262	65		4283	363	9661	441
	96.7	1496	72		4750	173	11,103	636
	98.8	1814	117		5864	915	12,742	1013
	99.7	1660	155		4590	393	12,162	746
**Marshall Samples**	95.8	1384	122		4404	312	11,075	771
	98.4	1857	137		5669	266	12,951	1226
	100	2086	166		6012	390	14,331	733
	100.3	2197	159		6201	334	14,446	958
